# Isolated Hypoglossal Nerve Palsy in the Setting of Concurrent Vertebral Artery Dissection and Internal Carotid Artery Dissection Plus Pseudoaneurysm: Case Report and Literature Review

**DOI:** 10.3390/brainsci15030225

**Published:** 2025-02-21

**Authors:** Cuong P. Luu, Benjamin Lee, Matthew E. Larson, Garret P. Greeneway, Mustafa K. Baskaya

**Affiliations:** 1 Wisconsin Institute for Translational Neuroengineering, University of Wisconsin-Madison, WIMR 2, 1111 Highland Ave., Madison, WI 53705, USA; cuong.luu@wisc.edu; 2 Department of Neurological Surgery, University of Wisconsin-Madison, 600 Highland Ave., Madison, WI 53792, USA; blee@uwhealth.org (B.L.); ggreeneway@uwhealth.org (G.P.G.); 3 Department of Radiology, University of Wisconsin-Madison, 600 Highland Ave., Madison, WI 53792, USA; mlarson4@uwhealth.org

**Keywords:** spontaneous pseudoaneurysm, craniocervical artery dissection, hypoglossal nerve palsy, case report

## Abstract

**Background:** In rare cases, isolated hypoglossal palsy may arise from dissection and/or pseudoaneurysm of either the internal carotid artery (ICA) or the vertebral artery (VA). However, the mechanism of this pathology has not been elucidated, and no high-quality randomized data exist to guide its management. **Case Description:** A 43-year-old man without a significant medical history presented with signs of isolated right hypoglossal palsy following a vigorous coughing episode. Imaging demonstrated dissection and pseudoaneurysm of the left ICA in addition to dissection of the right VA. After 2 weeks on 325 mg aspirin daily, the patient presented with left (rather than right) tongue symptoms and worsening ICA and VA stenosis. While on 325 mg aspirin plus 75 mg clopidogrel daily without additional endovascular intervention, the patient improved with no residual symptoms at 6 weeks from symptom onset. **Conclusions:** Acute hypoglossal nerve palsy may present with ipsilateral swelling, which could be mistaken for contralateral atrophy. We suggest ordering a CT angiogram initially to delineate a potential ICA versus VA dissection, as well as to rule out other etiologies. In our case, dissection and pseudoaneurysm from the ICA likely led to hypoglossal palsy through a mass effect on the nerve. Our comprehensive literature review favors initial management with dual-antiplatelet agents, and to then escalate to procedural interventions if symptoms worsen.

## 1. Introduction

While uncommon, craniocervical artery dissections may carry devastating neurological complications. Internal carotid artery (ICA) dissections present in less than 3 per 100,000 in the general population, with only 50% developing a pseudoaneurysm. Vertebral artery (VA) dissections occur even more rarely, at about 1 per 100,000 [1]. These dissections and pseudoaneurysms have a wide variety of etiologies, ranging from traumatic to idiopathic causes. Connective tissue diseases or infections further predispose patients to these conditions [2]. Although most cases present with ischemic stroke, rare cases of dissections and pseudoaneurysms in either the ICA or the VA present with isolated cranial neuropathy [3,4,5,6]. The isolated presentation makes diagnosis difficult. Skull base fractures, high cervical lymphadenopathy, primary and metastatic neoplasms, neurogenic infections, and vasculitis may also cause outwardly isolated cranial neuropathy. Furthermore, in cases where vascular dissection is the cause leading to cranial nerve paresis, it is unclear if the mechanism is from mass effect leading to nerve compression, or if there is a compromised blood supply to the nerve [4].

Herein, we present a very rare case of ICA dissection and pseudoaneurysm plus VA dissection, leading to an isolated hypoglossal nerve palsy. To the best of our knowledge, this case report describes the second youngest male ever reported to present with a carotid artery dissection/pseudoaneurysm and ipsilateral hypoglossal nerve palsy. Our primary objective is to present this rare case, synthesize findings with prior cases in the literature, and highlight key clinical considerations for this rare pathology.

## 2. Detailed Case Description

### 2.1. Initial Presentation

**Figure 1:**
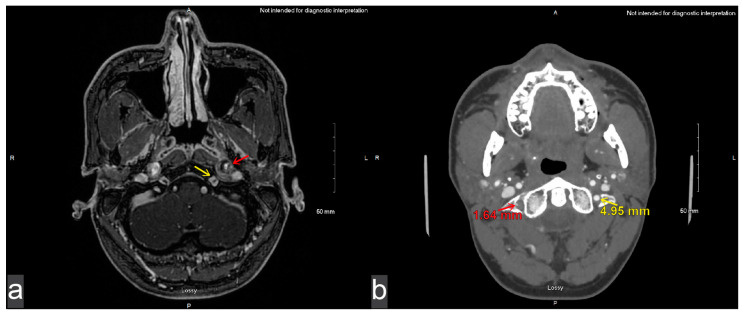
Dissections of the left internal carotid artery and right vertebral artery prior to hospitalization (Δ time indicates duration since symptom onset). (**a**) Postcontrast Axial T1 Bravo (Δ3 days): Dissection of the left ICA with pseudoaneurysm formation (red) adjacent to the hypoglossal canal (yellow). (**b**) Axial CTA (Δ3 days): Focal narrowing of right VA (red) at the C1 transverse foramen in comparison to the normal left VA (yellow). Congenitally dominant left VA.

**Figure 2:**
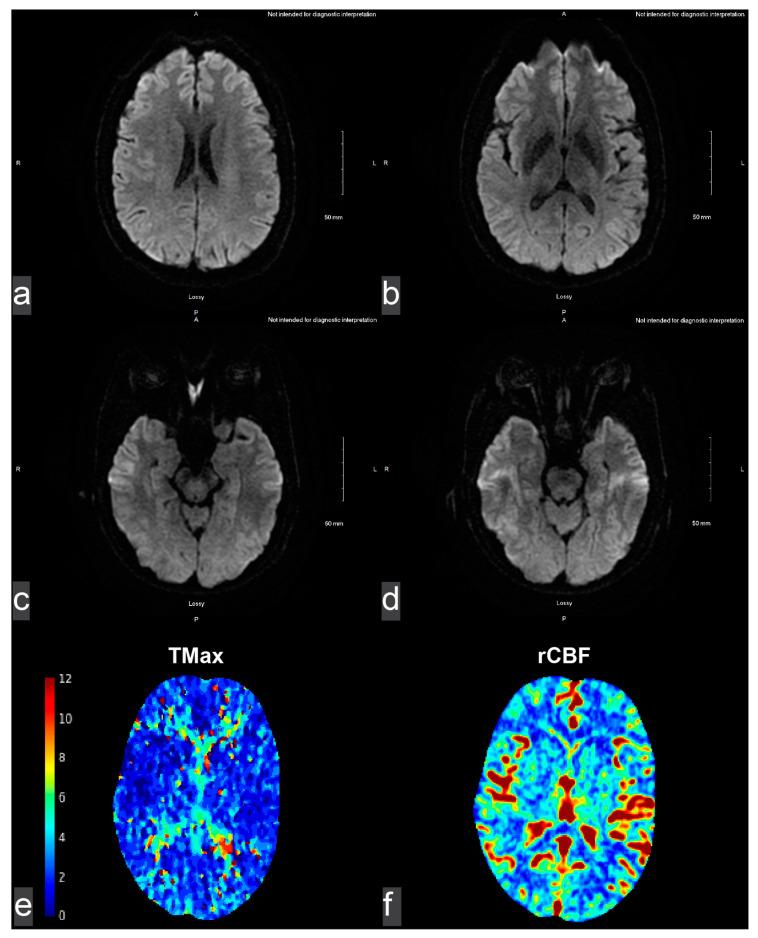
No evidence of cerebral flow deficit or ischemia despite dissections of the left internal carotid artery and right vertebral artery prior to hospitalization (Δ time indicates duration since symptom onset). (**a**–**d**) Axial DWI (b = 1000) (Δ3 days): No hyperintensity indicative of acute ischemic stroke along the (**a**) motor cortex, (**b**) posterior limb of the internal capsule, (**c**) crus cerebri, or (**d**) hypoglossal nucleus. (**e**,**f**) CT perfusion (Δ17 days): No markedly (**e**) prolonged Tmax or (**f**) decreased rCBF to indicate a significant limit in flow from the left ICA stenosis.

**Figure 3:**
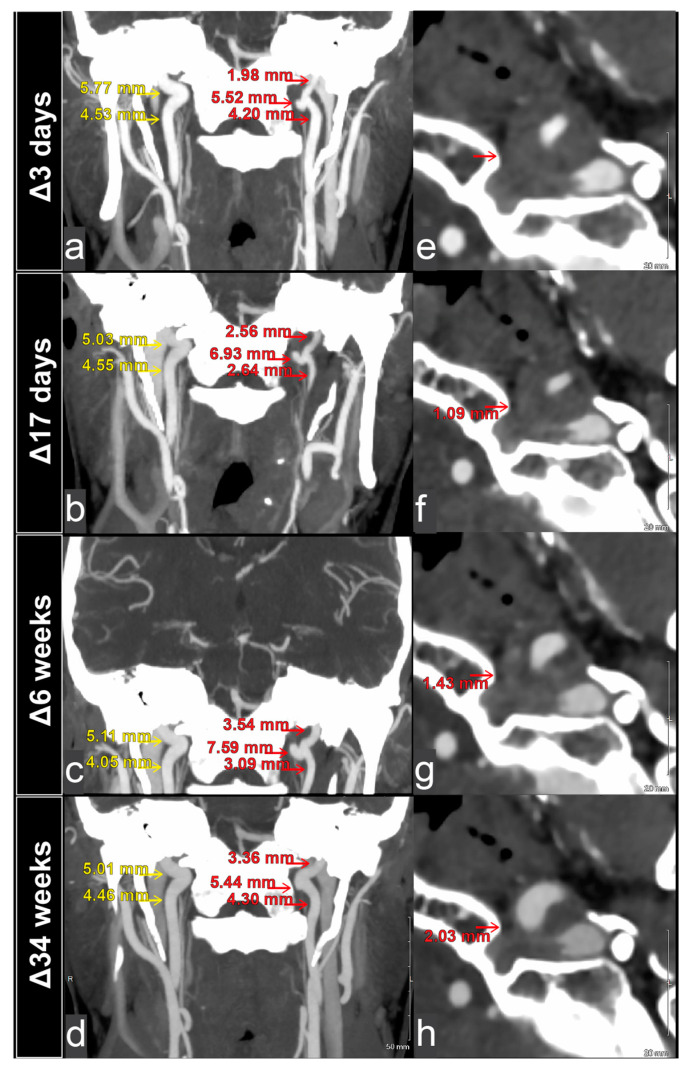
Progression of the pseudoaneurysm on left internal carotid artery and its mass effect on the left hypoglossal canal (red indicates the pathologic side; yellow indicates the normal side; Δ time indicates duration since symptom onset). (**a**–**d**) Coronal CTA showing left ICA dissection with luminal narrowing and pseudoaneurysm at the skull base at (**a**) Δ3 days, (**b**) Δ17 days, (**c**) Δ6 weeks, and (**d**) Δ34 weeks. Size of the pseudoaneurysm grew from Δ3 days to Δ6 weeks, before shrinking by Δ34 weeks. The growth at Δ17 days called for potential coiling, which we decided against. The lumen of the left ICA distal to pseudoaneurysm continued to expand at each sequential imaging session following the initial presentation (measurements indicate the widest diameter of the marked vessel or pseudoaneurysm). (**e**–**h**) Axial CTA showing the left ICA dissection (**e**) with mass effect at the hypoglossal canal at Δ3 days, followed by a progressive increase in space with likely fat infiltration at (**f**) Δ17 days, (**g**) Δ6 weeks, and (**h**) Δ34 weeks. The mass effect suggests compression and subsequent relief of the hypoglossal nerve over weeks, correlating with the slow and gradual improvement in the patient’s symptoms. (Anatomical levels matched based on cortical and trabecular bone landmarks to provide relative estimates of the diameter of the fat infiltration areas [poorly attenuating black spots]).

A 43-year-old man presented to the emergency department with 3 days of abnormal tongue movement, tongue ache, and slurred speech. He reported 2 weeks of significant coughing, mild neck pain, and mild headache that had resolved around the onset of the tongue symptoms. He denied recent trauma. His medical history was specifically unremarkable for cardiovascular disease, prior vascular dissection, or known connective tissue disease. Surgical history was only notable for wisdom teeth extraction. The patient denied current tobacco use and reported 7.0 standard drinks of alcohol per week. Family history was also unremarkable for cardiovascular disease, prior vascular dissection, and known connective tissue disease. BMI was 29.3 kg/m^2^ and blood pressure was 132/99 mmHg. Upon physical examination, mild right-sided tongue atrophy, right tongue deviation on protrusion, and dysarthric speech were notable. The remainder of his neurological examination was unremarkable. Given that his lateralizing signs were concerning for stroke, he was sent for initial imaging with MRI (magnetic resonance imaging) and CTA (computed tomographic angiography). The MRI revealed a thickening of the left cervical internal carotid artery, along with a pseudoaneurysm near the hypoglossal canal (Figure 1a). There were no diffusion-weighted imaging changes to suggest infarct. The CTA demonstrated significant stenosis of the right vertebral artery (Figure 1b). Despite the stenoses, cerebral perfusion remained normal with no sign of penumbra or ischemia (Figure 2). Further study of the vessels via CTA showed 50% focal stenosis of both the left ICA at the upper cervical to petrous segment (Figure 3a) and the right vertebral artery at the level of the transverse foramen. The CTA additionally highlighted a 5.52 by 5.00 mm skull-base pseudoaneurysm forming from the left ICA dissection (Figure 3a). The patient was discharged on 325 mg aspirin per day.

### 2.2. Two-Week Follow-Up Visit

The patient was seen at a two-week follow-up visit at the neurosurgery clinic. Upon physical examination, mild dysarthria of the “TH” sound, along with newly found left, rather than right, tongue deviation on protrusion and weak tongue push on the left cheek were notable. Repeated CTA scans of the left ICA showed worsening stenosis from 50 to 70% and an expanding pseudoaneurysm (6.93 by 6.50 mm) at the skull base (Figure 3b). The worsening left ICA findings prompted a formal diagnostic cerebral angiogram, which confirmed a stable dissection and pseudoaneurysm formation proximal to the petrous section of the left ICA. No intervention was performed. Concurrent workups for other etiologies, such as vasculitis (primary central nervous system, ANCA-associated, systemic lupus erythematosus, Behcet, Sjogren, cryoglobulinemia, and antiphospholipid syndrome) and infections (HIV, syphilis, HSV, and VZV), yielded unremarkable results. The patient was discharged home on 325 mg aspirin plus 75 mg clopidogrel daily (dual antiplatelet therapy, DAPT).

### 2.3. Six-Week Follow-Up Visit

In the following month (Δ6 weeks), CTA scans showed an improving luminal caliber of the left ICA and right VA. However, the skull base pseudoaneurysm on the left ICA grew to 7.59 by 5.51 mm (Figure 3c). Despite this, axial CTA scans began to show increased spacing between the pseudoaneurysm and the hypoglossal canal relative to the previous two presentations (Figure 3g vs. Figure 3e,f). The patient concurrently denied tongue symptoms.

### 2.4. Six-Month Follow-Up Visit

After 6 more months (Δ34 weeks), CTA scans showed continued improvement in the caliber of the left ICA and right VA. The pseudoaneurysm shrunk to 5.44 by 5.84 mm (Figure 3d). The area of fat infiltration by the left hypoglossal canal continued to expand (Figure 3h). On repeated physical examination, the tongue was midline. The patient was kept solely on 325 mg aspirin per day.

The patient remains without new or additional tongue complaints at over one year (Δ52 weeks).

## 3. Discussion

Case Summary: Our patient presented with isolated hypoglossal nerve palsy in the setting of dissection and pseudoaneurysm of the left ICA plus dissection of the right VA. Dysarthria and tongue incoordination persisted for at least Δ17 days following onset and were resolved by Δ6 weeks. Resolution occurred while on 325 mg aspirin and 75 mg clopidogrel daily without endovascular intervention.

**Figure 4:**
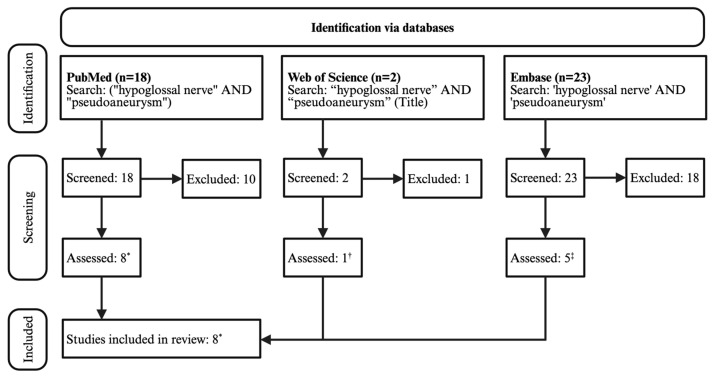
Literature review of PubMed, Web of Science, and Embase for spontaneous pseudoaneurysm and concurrent hypoglossal nerve palsy. Included study references: * [1,5,9,10,13,17,18,19]; † [1]; ‡ [1,5,9,13,17].

Literature Review: We questioned if and how spontaneous dissection and/or pseudoaneurysm may have progressed and caused hypoglossal nerve palsy in prior reports. We thus performed a comprehensive literature review on PubMed, Web of Science, and Embase, with results reported following the Preferred Reporting Items for Systemic Reviews and Meta-analyses guidelines [7,8]. On PubMed, we searched (“hypoglossal nerve” AND “pseudoaneurysm”), which we then translated to equivalent phrases for searches using Web of Science and Embase (Figure 4). We included (1) full-length, peer-reviewed journal articles describing (2) the ICA or VA, (3) spontaneous pseudoaneurysm, and (4) concurrent hypoglossal palsy. We excluded articles that may have covered ICA or VA dissection but (1) without pseudoaneurysm and (2) reports that were not in English. Data points collected included age and sex, smoker status, vascular defect and location, symptoms and examination at presentation, imaging, vascular management, and residual symptoms. Data were reported in their entirety as seen in each case, with minor processing for consistent language and formatting among articles.

Our literature review of the three databases on 14 December 2023 found eight additional case reports of spontaneous ICA pseudoaneurysm with concurrent hypoglossal palsy (Figure 4) [9,10,11,12,13,14,15,16]. The analysis of nine cases, including our own, reveals several patterns that may inform pathophysiological understanding, diagnostic approaches, and management strategies (Table 1).

Disease presentation: The marked male predominance (eight of nine cases) and left-sided prevalence (seven of nine cases) suggest potential anatomical or physiological factors that may predispose certain populations to this condition.

Since our patient did not have major trauma, his prior episode of severe, prolonged coughing may have been a trigger for the dissection of the left ICA and right VA. Craniocervical arterial dissections occur more frequently in the extracranial space where the ICA and VA have greater mobility and may rub against bony structures [20]. This connection has been reinforced in at least three additional cases of healthy, middle-aged men who developed carotid artery dissections after prolonged coughing, including the case presented by Evan et al. in Table 1 [12,21,22]. Moreover, the left-sided predominance of ICA dissections, found in our review, may relate to the direct origin of the left carotid from the aortic arch, creating higher mechanical stresses compared to the right carotid’s origin from the brachiocephalic artery. This suggests the continued need to find an effective method of cough suppression, both to improve patient comfort and to reduce the rare case of vascular injury. This also raises the importance of preventing long-term structural damage, such as that from smoking, to reduce the potential vulnerability of the vessel to physical agitation [23]. The frequent presentation of ICA dissection and pseudoaneurysm in middle-aged males could indicate a late-term effect of smoking, though smoking data are not sufficiently well reported in Table 1 to corroborate this hypothesis. Alternatively, smoking may bias the development of true aneurysms rather than pseudoaneurysms of the ICA. Epidemiological data from the Swedish National Registry for Vascular Surgery during 1997 to 2011 reported that 39% of 36 aneurysm patients had smoked, while 0% of 12 pseudoaneurysm cases had smoked [24]. In either scenario, with one in three high schoolers using e-cigarettes and with e-cigarettes linked to endothelial injury and vascular stiffening, further study of the correlation between ICA aneurysm or pseudoaneurysm and e-cigarette use is warranted [25].

Besides a coughing history, signs of craniocervical artery pseudoaneurysm to be aware of include pain in the head, face, or neck (as with our patient), Horner Syndrome, pulsatile tinnitus, cranial nerve(s) palsy, and ischemic stroke (compiled in Table 2). Lower cranial nerve palsy occurs in about 5% of cases, with the variable involvement of cranial nerves IX, X, XI, and XII [4,26].

Of note, our patient’s initial clinic presentation was notable for right tongue atrophy and right-ward tongue deviation, indicating possible right hypoglossal nerve palsy. However, upon re-examination by the neurosurgical team during consultation and hospitalization, a left tongue deviation and a weak left tongue push were notable, supporting a left hypoglossal nerve palsy [17]. Both exams had credibility because the patient suffered both a left ICA pseudoaneurysm and a right VA dissection. Furthermore, there is a wide differential for potential mechanisms (vascular, infectious, neoplastic, inflammatory, etc.) that may cause isolated hypoglossal nerve palsy. Of note, hypoglossal dysfunction may also arise from ischemic damage, mechanical compression, and/or a combination of the two. In our case, the left ICA pseudoaneurysm could have caused a left hypoglossal nerve palsy via mechanical compression. In contrast, the right VA dissection in our case also could have led to a posterior circulation deficit and subsequent right hypoglossal nucleus palsy via ischemic damage. The latter mechanism is admittedly less common: 57–84% of VA dissections cause posterior circulation deficit, with hypoglossal nucleus palsy only occurring in very rare cases [3,27]. Here, DWI MRI and perfusion CT scans were unremarkable for any evidence of ischemia, specifically in the superior posterior right medulla, where the hypoglossal nucleus is often located (Figure 2). Additionally, the CTA demonstrated the abutment of the left ICA pseudoaneurysm on the left hypoglossal canal, which likely led to mass effect on the left hypoglossal nerve. These contradictory examination findings may be explained by transient edema of the hemitongue ipsilateral to the hypoglossal paresis. Transient swelling manifests acutely within 48 h of nerve paresis as fluid shifts to the extracellular space and/or as the capillary bed enlarges. In contrast, fatty atrophy of the lingual muscles occurs with more chronic denervation (Table 1) [13,15]. The asymmetric swelling ipsilateral to the side of the hypoglossal palsy may have led to the perception of relative atrophy and the deviation of the non-affected, contralateral hemitongue. These examination errors were also reported in the case by Evan et al. [12]. Thus, physicians should be wary of the time scale of swelling versus atrophy following denervation to reduce bias upon examination. This would help prevent potential diagnostic confusion, miscommunication, and mismanagement.

Disease Progression: The temporal progression of symptoms also emerged as a crucial diagnostic consideration from our review. Most cases demonstrated a characteristic evolution: early presentation with ipsilateral tongue swelling and deviation (typically within days), followed by eventual atrophy in untreated cases (developing over months).

When the patient noted no residual symptom at his visit Δ6 weeks from symptom onset, the CTA concurrently showed an increase in fat infiltration adjacent to the hypoglossal canal (Figure 3g). This soft tissue infiltration likely made more space in the area and relieved pressure on the exiting hypoglossal nerve. The correlation between the imaging and symptoms at sequential visits provides corroborating evidence that hypoglossal nerve palsy originated from direct compression in our patient’s case. If the hypoglossal nerve’s blood supply was compromised due to ICA dissection, this would require the patient to have an uncommon anatomy: an anomalous ascending pharyngeal artery originating from the ICA bifurcation (rather than from its typical source, the external carotid artery) [4,28]. More, the resulting compromise would cause ischemic damage, and tongue function would likely not return to baseline following ischemic damage; however, this runs contrary to the recovery seen in most patients in our review (Table 1).

Diagnostic Imaging: Table 1 shows that CT, CTA, MRI, MRA, and DSA have been used to diagnose craniocervical artery dissections. DSA is considered the gold standard, but it is more invasive than the other modalities. DSA is also more likely to miss a pseudoaneurysm formation [27]. For diagnosing internal carotid dissection, MRI (84% sensitivity, 99% specificity) and MRA (95% sensitivity, 99% specificity) perform very well. However, both perform poorly (<60% sensitivity, <60% specificity) in diagnosing vertebral artery dissection. In contrast, CTA provides high performance for diagnosing ICA dissection (>95% sensitivity, >95% specificity), while also excelling at tracking VA dissection (74–98% sensitivity, 84–100% specificity) [29]. Though CT and MRI are worse at diagnosing vascular dissections, these non-angiographic imaging methods still serve a purpose for tracking sequalae, such as cerebral ischemia, muscular edema, and atrophy. In our case, we initially ordered the MRI and CTA to identify ischemic stroke and vascular defects. We recommend ordering a set of MRI and CTA scans for future presentations that may originate from multiple alternative vascular injuries, such as ICA or VA dissections.

Management: Management approaches across the reviewed cases demonstrated varying levels of intervention, from conservative medical therapy to endovascular treatment. Of the eight cases with reported management, five achieved symptom resolution with antiplatelet or anticoagulation therapy alone, while three proceeded with endovascular intervention. Among the five cases managed conservatively with antithrombotics alone, four achieved complete symptom resolution within six months.

In general, the management of craniocervical artery dissection often begins with antiplatelet or anticoagulation approaches to lower the risks of thrombosis, embolism, and ischemic stroke [30]. However, it is best to avoid using anticoagulants when there is a concurrent intracranial (pseudo-)aneurysm, as anticoagulation increases the risk of subarachnoid hemorrhage [18]. When an extracranial dissection presents with a pseudoaneurysm, endovascular or surgical intervention may be indicated if neurological symptoms remain refractory to medical management and/or the dissection or pseudoaneurysm continue to expand [27]. Admittedly, these indications for endovascular or surgical intervention are rare. Endovascular interventions typically involve balloon angioplasty, followed by the placement of one or more stents. Surgical interventions (i.e., bypass) are rarely indicated and/or performed to treat such pathologies. In our patient, his persistent tongue symptoms and worsening stenosis led us to perform a diagnostic cerebral angiogram to fully evaluate the intricacies of the dissection and pseudoaneurysm. Fortunately, his cerebral angiogram showed stable dissections and a pseudoaneurysm. This convinced us against stenting or embolization. Overall, however, there are no insightful, randomized studies on the treatment of carotid dissections and pseudoaneurysms. This may be because of typically benign progressions at follow-ups, as seen in five of the eight cases with only antiplatelet or anticoagulation treatments (Table 1). Large aggregate outcome studies would help confirm or deny this trend and guide further research, if needed.

While not administered in our patient, another aspect of conservative treatment that should be considered for cranial neuropathy is short-term systemic corticosteroids. There is a paucity of studies in the literature that specifically analyze the use of systemic corticosteroids in patients with cranial neuropathy secondary to similar vascular pathologies; future studies could further analyze the role and outcomes of systemic corticosteroids in patients with this presentation. Currently, clinicians should weigh the risks and benefits of systemic corticosteroid treatment on a case-by-case basis.

Our patient experienced an improvement in hypoglossal palsy symptoms by Δ6 weeks from symptom onset. Hypoglossal palsy symptoms from spontaneous ICA pseudoaneurysm may still be present up to three months after onset, though most cases reported a complete resolution in the months after (Table 1). Most recurrences occur within the first four–six weeks of symptom onset, involving the dissection of arteries in series with the previous injury [31]. After one month, the recurrence risk is reportedly only 1% per year [19].

Our successful conservative management of concurrent dissections adds to the limited literature on this topic and suggests that, even with multiple vessel involvement, DAPT may be adequate when close monitoring is maintained. Acknowledging the limitations from the aggregated case reports [32], we propose a management algorithm for similar cases until further studies are conducted: (1) initial DAPT, (2) close radiological monitoring with CTA if symptoms persist or worsen, and (3) consideration of endovascular intervention only if angiogram confirms progression or patient shows clinical deterioration despite optimal medical therapy.

## 4. Conclusions

Acute hypoglossal nerve palsy may present with asymmetric swelling that may mislead one to perceive atrophy and deficit on the contralateral hemitongue without paresis. Initial work-up in such patients needs to prioritize CTA, as CTA is more likely to reveal both ICA and VA dissections compared to MRA. CTA may also demonstrate the presence of mass effect on the hypoglossal canal, a likely cause of hypoglossal nerve palsy. Additionally, CTA provides a rapid rule-out for embolic stroke. Management should begin with DAPT to lower the risk of embolic ischemia and, only then, escalate to procedural interventions if symptoms worsen.

## Figures and Tables

**Table 1:** Reported cases of internal carotid artery pseudoaneurysms and hypoglossal nerve palsy.

	Author (Year)	Age (Sex)	Smoking (Pack-Years)	Vascular Defect *	Presentation (Duration at Index Visit and Symptoms)	Imaging	Vascular Management	Residual Symptoms (Follow-Up Time in Months)
1	Pumar et al. (1992) [14]	57 (M)	ND	Right ICA pseudoaneurysm	6 mo: right tongue incoordination and atrophy, right palatal arch numbness, dysphagia, submandibular swelling	MRI DSA	ND	ND (ND)
2	Ursekar et al. (2000) [16]	37 (F)	ND	Left ICA pseudoaneurysm	1 wk: transient ischemic attack, headache, left jaw pain, dysarthria	MRI DSA	Daily heparin, then 6 weeks oral anticoagulation	Tongue movement improved (1.5)
3	Kaushik et al. (2009) [13]	44 (M)	ND	Left ICA dissection and pseudoaneurysm	2 wk: dysarthria 2 d: tongue swelling, dyspnea	MRI/MRA	Heparin and warfarin	Asymptomatic (ND)
4	Stubgen (2011) [15]	56 (M)	ND	Left ICA dissection and pseudoaneurysm	6 wk: left tongue swelling, tongue biting, dysarthria 1 wk: left tongue weakness, palatal arches asymmetry	MRI	Antiplatelet regimen	Asymptomatic (12)
5	Cruciata et al. (2017) [10]	56 (M)	ND	Left ICA dissection and pseudoaneurysm	Days: dysphagia, elevated blood pressure (170/90 mmHg)	MRI/MRA	Steroids and low-molecular-weight heparin, coil embolization	Dysphagia improved (2)
6	English et al. (2018) [11]	48 (M)	ND	Left ICA dissection and pseudoaneurysm	3 d: headache, sore throat, malaise 1 d: left tongue weakness, dysphagia, dysarthria, left arm incoordination	MRI CTA	Interval follow-ups	Asymptomatic (5)
7	Evan et al. (2021) [12]	42 (M)	0	Right ICA dissection and pseudoaneurysm	1 wk: sore throat, sinus congestion, heavy non-productive coughing, right tongue swelling, right tongue deviation, left uvula deviation, right vocal cord paralysis, dysphagia, dysarthria, posterior neck swelling	CT MRI DSA	Aspirin and clopidogrel, coil embolization	Dysarthria improved; residual tongue movement, and palatal deviation (2); dysphagia resolved (3)
8	Abukeshek et al. (2022) [9]	56 (M)	20	Left ICA dissection and pseudoaneurysm	1.5 mo: left tongue atrophy, left tongue deviation, poor mastication, dysphagia, dysarthria	MRI/MRA	Endovascular stenting	Dysarthria resolved (3); poor mastication, dysphagia (3)
9	Present Case (2023)	43 (M)	4.5	Left ICA dissection and pseudoaneurysm, right VA dissection	3 d: tongue deviation, dysarthria	MRI CTA Angiography	325 mg aspirin plus 75 mg clopidogrel daily	Asymptomatic (1.5)

* All vascular pathologies occurred below the skull base. CT: computed tomography; CTA: computed tomographic angiography; d: day(s); DSA: digital subtraction angiography; F: female; ICA: internal carotid artery; M: male; mo: month(s); MRA: magnetic resonance angiography; MRI:f magnetic resonance imaging; ND: no data; VA; vertebral artery; wk: week(s).

**Table 2:** Internal carotid artery pseudoaneurysm—a clinical summary.

Demographics	- 1.5 per 100,000 - Male sex
Etiology and Risk Factors	- Traumatic or spontaneous trigger - Vasculitis, infection, and connective tissue disease
Presentation	- Head, facial, or neck pain - Horner syndrome - Pulsatile tinnitus - Cranial nerve(s) palsy - E.g., hypoglossal palsy with ipsilateral tongue deviation and swelling (days) or atrophy (month) - Ischemic stroke
Diagnosis	- Imaging: - CTA - MRI (ruling out ischemic changes) - Rule out other etiologies: vasculitis, infection, and connective tissue disease
Treatment	Initial DAPT Close radiological monitoring with CTA if symptoms persist or worsen Consider endovascular intervention only if angiogram confirms progression or patient shows clinical deterioration despite optimal medical therapy
Prognosis	- May resolve without residual symptoms in months - Most recurrences occur within 4-6 weeks of symptom onset

## Data Availability

All relevant original data are as presented in this study. Further inquiries can be directed to the corresponding author.

## Abbreviations

The following abbreviations are used in this manuscript:

ANCA	anti-neutrophil cytoplasmic antibody
BMI	body mass index
CT	computed tomography
CTA	computed tomography angiography
d	day(s)
DAPT	dual antiplatelet therapy
DSA	digital subtraction angiography
DWI	diffusion-weighted imaging
F	female
HIV	human immunodeficiency virus
HSV	herpes simplex virus
ICA	internal carotid artery
M	male
mo	month(s)
MRA	magnetic resonance angiography
MRI	magnetic resonance imaging
ND	no data
VA	vertebral artery
VZV	varicella zoster virus
wk	week(s)
yr	year(s)
